# Advances in understanding the reproductive toxicity of endocrine-disrupting chemicals in women

**DOI:** 10.3389/fcell.2024.1390247

**Published:** 2024-03-28

**Authors:** Jinguang Wang, Chunwu Zhao, Jie Feng, Pingping Sun, Yuhua Zhang, Ailing Han, Yuemin Zhang, Huagang Ma

**Affiliations:** ^1^ Reproductive Medicine Center of Weifang People’s Hospital, Weifang, China; ^2^ Gastrointestinal Surgery Center of Weifang People’s Hospital, Weifang, China; ^3^ Gynecology and Obstetrics Department, Fangzi District People’s Hospital, Weifang, China

**Keywords:** environmental pollution, endocrine disrupting chemicals, reproductive health, reproduction toxicity, exposure

## Abstract

Recently, there has been a noticeable increase in disorders of the female reproductive system, accompanied by a rise in adverse pregnancy outcomes. This trend is increasingly being linked to environmental pollution, particularly through the lens of Endocrine Disrupting Chemicals (EDCs). These external agents disrupt natural processes of hormones, including synthesis, metabolism, secretion, transport, binding, as well as elimination. These disruptions can significantly impair human reproductive functions. A wealth of animal studies and epidemiological research indicates that exposure to toxic environmental factors can interfere with the endocrine system’s normal functioning, resulting in negative reproductive outcomes. However, the mechanisms of these adverse effects are largely unknown. This work reviews the reproductive toxicity of five major environmental EDCs—Bisphenol A (BPA), Phthalates (PAEs), Triclocarban Triclosan and Disinfection Byproducts (DBPs)—to lay a foundational theoretical basis for further toxicological study of EDCs. Additionally, it aims to spark advancements in the prevention and treatment of female reproductive toxicity caused by these chemicals.

## 1 Overview of research on endocrine disruptors

In recent years, the relentless advance of industrialization has dramatically escalated environmental pollution, casting a long shadow over public health. A significant and growing body of evidence has illuminated the detrimental effects of endocrine disrupting chemicals (EDCs) on female reproductive health, including several conditions such as infertility (Karwacka et al., 2019). EDCs, a class of exogenous chemicals, disrupt the natural hormone synthesis and metabolic processes in organisms, triggering a cascade of adverse outcomes, as well as impacting embryogenesis and fetal development (Dutta et al., 2023). The ubiquity of human exposure to these environmental pollutants is a pressing concern, occurring through ingestion, inhalation, or dermal absorption (Yilmaz et al., 2020). Research underscores that adverse environmental influences across the entire spectrum of a woman’s life can exert profound and enduring effects on reproductive health (Ma et al., 2019; Panagopoulos et al., 2023).

Based on the prevalence and ubiquity, emerging concerns and the relevance to female reproductive health, this article embarks on a comprehensive review of five pivotal EDCs: Bisphenol A (BPA), Phthalic acid esters (PAEs), Triclocarban (TCC), Triclosan (TCS), and Disinfection byproducts (DBPs). (Table 1). These compounds were chosen due to their widespread presence in the environment and their high potential for human exposure. For instance, BPA is commonly found in plastics and resins, whereas PAEs are prevalent in various consumer products, including personal care items and plastics. Some of these chemicals, such as TCC and TCS, represent emerging contaminants of concern. Their inclusion reflects the evolving nature of environmental health research and the need to address less studied, but potentially significant, threats. Each of these chemicals has been linked to various degrees of interference with hormonal function, underscoring the need for a nuanced understanding of their toxicological profiles. This review not only lays a foundational theoretical framework for the ongoing study of EDCs but also aims to shed light on potential strategies for mitigating female reproductive toxicity and fostering preventive health measures.

**TABLE 1:** Summary of female reproductive toxicity of endocrine-disrupting chemicals in human and animal.

Chemical	Human	Animal
Bisphenol A	↓Number of ovarian follicles, mature oocytes, and fertilization rate	↓ Number of primordial follicles, preantral follicles, and antral follicles
	↓Granulosa cell aromatase expression in PCOS patients	Meiotic division defects in mouse oocytes
	↑Expression of endometrial ER promotes EMT development	Lipid peroxidation and free radical generation lead to cell apoptosis
	↓GnRH	Disruption of steroidogenesis and interference with the aromatase receptor pathway
	Affecting HPO axis function, disrupting gonadal and gamete function	Dysregulation of cell cycle regulators
		Abnormal proliferation of endometrium, affecting embryo implantation
PAEs	↑ Miscarriage rate	↓ Quality of mouse oocytes, embryonic development capability
	↓ Oocyte retrieval, MII rate, pregnancy rate, and live birth rate	↓ Hormone secretion and lowers expression of steroidogenic enzymes
	Affects female reproductive prognosis and ovarian function	Affects organelles and epigenetic modifications in mouse oocytes to diminish their maturation and fertilization ability
	Associated with metabolic disturbances in PCOS patients	Disrupts ovarian function, leading to extended estrous cycles
	Alters signal transduction pathways affecting the occurrence and development of EMT	
TCC/TCS	↑ Risk of fetal malformations	↑ Mortality and malformation rates in zebrafish embryos
	↓ Gestational age	↓ Hatching and shortened body length
	↓ Fertility	↓ Progesterone biosynthesis may disrupt the implantation process
	Disruption of the biosynthesis of steroid hormones and the dynamic balance of hormones	Abnormal embryonic development in zebrafish
		Induction of early embryonic epigenetic modifications, leading to oxidative stress and DNA damage in mouse early embryos
DBPs	↑ Risk of preterm birth, stillbirth, low birth weight, and adverse pregnancy outcomes with birth defects	↓ Hatching rate, reduced tail length, and increased malformation rate in zebrafish
	↓ Ovarian reserve, oocyte maturation, fertility, and embryo development	↑ ROS levels, disruption of organelles, affecting mouse oocyte maturation
	Causes changes in reproductive hormone levels, menstrual cycle disturbances	Affects HPO axis function, inhibits preantral follicle growth, lowers E2 levels, causing ovarian toxicity
		Impacts rat fertility, pre-conception survival rate, and postnatal survival rate

## 2 Reproductive toxicity of bisphenol A

As a prevalent component of the bisphenol (bis-hydroxyphenyl) group, Bisphenol A (BPA) is extensively utilized in the manufacturing of high molecular weight materials, such as polycarbonates and epoxy resins (Hananeh et al., 2021). Its widespread application in food and beverage packaging, baby bottles, safety equipment, and medical devices has raised concerns (Abraham and Chakraborty, 2020). There are various ways in which humans are exposed to BPA, with oral exposure being the primary route, although it can also be inhaled through the respiratory tract or percutaneous absorption (Sonavane and Gassman, 2019). BPA exists in various biological samples, including blood, urine, breast milk, umbilical cord blood, amniotic fluid, and placental tissue, among others (Zhang et al., 2020). BPA undergoes metabolism in the form of glucuronide and sulfate conjugates, with a biological half-life of approximately 6 h (Ougier et al., 2021). Currently, the United States Environmental Protection Agency sets the maximum acceptable dose of BPA at 0.05 mg/kg.bw, but the actual doses that humans are exposed to may be higher, raising widespread concern among researchers and the general public.

### 2.1 Epidemiological research

In recent years, increasing evidence suggests that BPA has adverse effects on the female reproduction. The molecular structural characteristics of BPA give it a “weak” estrogenic effect, allowing it to selectively bind to estrogen receptors (ER) subtypes, activate ER-dependent signaling pathways, and regulate cell growth-related genes expression (Wang et al., 2021). BPA has been shown to be associated with the onset of various endocrine disorders, including female fertility, polycystic ovarian syndrome (PCOS), and endometriosis, affecting the morphology and function of the Hypothalamic-Pituitary-Ovary (HPO) axis (Stavridis et al., 2022). Regulation of cell growth-related gene expression.

In women undergoing assisted reproductive treatment, a significant correlation has been observed between elevated urinary BPA levels and reduced antral follicle count, decreased oocyte quantity, lower fertilization rates, and implantation failures (Pivonello et al., 2020). Furthermore, PCOS women exhibit significantly higher total serum BPA exposure compared to the control group (PCOS: 167.04 ± 9.44 IU/mL, control: 31.94 ± 3.57 IU/mL), suggesting that higher BPA level in the follicular fluid of PCOS patients (PCOS: 440.50 ± 63.70 pg/mL, control: 338.00 ± 57.88 pg/mL) may play a crucial role in its pathogenesis by reducing aromatase expression in granulosa cells (Wang et al., 2017; Prabhu et al., 2023). Epidemiological meta-analyses have found an association between endometriosis development and BPA exposure due to its influence on epigenetics (Xue et al., 2021). BPA exposure can also directly or indirectly impact the HPO axis, disrupting gonadal and gamete functions, reducing the secretion of gonadotropin-releasing hormone (GnRH), subsequently decreasing ovarian steroid production, inhibiting ovulation, and leading to infertility (Santoro et al., 2019). Additionally, BPA can traverse the placental barrier to enter the amniotic fluid, leading to higher risks of macrosomia and prematurity (Loukas et al., 2023).

### 2.2 Animal experimental research

BPA is an ovarian toxicant that may exert its effects through multiple pathways, including lipid peroxidation, oxidative stress, and apoptosis (Mukherjee et al., 2024). In animal models, BPA reduces fertility, decreases the number of follicles, induces premature ovarian failure, and disrupts steroidogenesis.

In mice model, Hunt et al. (Patel et al., 2017) first reported that exposure to BPA during prenatal development led to the suppression of germ cell breakdown in F1, increased estradiol levels, and decreased the number of primordial, primary, and antral follicles on postnatal day 21. Furthermore, BPA exposure can lead to elevated urinary BPA levels, precocious puberty, altered estrous cycles, and changes in hypothalamic Kiss1 methylation status in F1 and F2 (Huang et al., 2022). Prolonged exposure to low doses of BPA inhibits the activation of ERα-induced AVPV-kisspeptin neurons, resulting in extended estrous cycles and reduced ovulation in adult female mice (Tang et al., 2020). BPA exposure can lead to meiotic division defects in mouse oocytes by disrupting gap junction communication between oocytes and granulosa cells and regulating the expression of cell cycle protein B1 in GVBD oocytes (Li et al., 2023). However, BPA-induced inhibition of follicle growth is likely mediated by its interruption of steroidogenesis, interference with the aromatase receptor pathway, and dysregulation of cell cycle regulators (Pan et al., 2021). BPA can dose-dependently regulate the function of granulosa cells in mouse ovarian preantral follicles and may impact pregnant mice and female offspring (Liang et al., 2021). Perinatal exposure of female rats to BPA results in early puberty onset, significantly thinner uterine lining, and activation of inflammation and aberrant autophagy in the offspring through TLR4/NF-κB and mTOR signaling pathways (Meng et al., 2020). Long-term exposure to BPA impairs the PGR-HAND2 pathway, leading to abnormal proliferation of uterine epithelium and adverse effects on embryo implantation and pregnancy (Li et al., 2016).

While the body of evidence linking BPA exposure to reproductive health issues continue to grow, inconsistencies in research findings necessitate further investigation. The observed PCOS-like abnormalities and endometriosis pathogenesis associated with BPA exposure, alongside adverse outcomes in assisted reproductive technologies, highlight the urgent need for comprehensive studies. Future research should focus on elucidating the complex interactions between BPA exposure and female fertility, considering the variability in exposure levels, biological responses, and environmental factors. Addressing these research gaps will be pivotal in developing targeted strategies to mitigate BPA’s reproductive toxicity and safeguard female reproductive health.

## 3 Reproductive toxicity of Phthalic acid esters

Phthalic acid esters (PAEs), commonly recognized as plasticizers, are integral to enhancing the flexibility and durability of plastic polymers (Paluselli and Kim, 2020). PAEs include high-molecular-weight compounds such as diisodecyl phthalate (DIDP) and diisononyl phthalate (DINP); intermediate compounds like butyl benzyl phthalate (BBP) and di(2-ethylhexyl) phthalate (DEHP); and low-molecular-weight compounds including diethyl phthalate (DEP) and di-n-butyl phthalate (DAP) (Kıralan, 2020). PAEs contaminate the air, soil, and natural water, and exposure to PAEs can occur through ingestion, inhalation, or skin absorption (Wang et al., 2023). The biological half-life of PAEs is short (<24 h), and the rapid biotransformation of PAEs into biologically active monoesters, which exhibit greater toxicity than their parent compounds, raises concerns regarding their short-term effects and long-term implications on human health, particularly in the realm of female reproductive health. (Zhang et al., 2021; Puri et al., 2023).

### 3.1 Epidemiological research

#### 3.1.1 Ovarian function and oocyte quality

PAEs have been identified as potential endocrine disruptors, capable of significantly impacting female reproductive health through the alteration of hormone levels and endogenous steroid hormone signaling. Exposure to PAEs can adversely affect hormone levels, affecting the female reproduction and even leading to adverse pregnancy outcomes (Zhang et al., 2023). Through the assessment of the correlation between PAEs metabolite concentrations in urine and assisted reproductive outcomes, it has been observed that decreased oocyte yield, reduced MII rates, lower clinical pregnancy rates, increased risk of implantation failure, and decreased live birth rates are all associated with PAEs exposure (Hauser et al., 2016). PAEs and their metabolites disrupt ovarian function by reducing the production of 17β-estradiol and inhibiting the growth of antral follicles. Messerlian et al. found that lower antral follicle counts were associated with higher PAEs exposure, and as metabolite concentrations increased, the mean antral follicle count significantly decreased (Messerlian et al., 2016). Evidence suggested that DEHP affect female reproductive prognosis and ovarian function (Yi et al., 2023). However, the connection between PAEs exposure and polycystic ovary syndrome (PCOS) remains controversial. Akın et al. assessed the correlation between PAEs and PCOS, finding that DEHP and DBP levels were significantly higher in PCOS patients than in the control group, suggesting an association between PAEs and PCOS (Akın et al., 2020). On the other hand, Akgül et al.’s study found no correlation between DEHP and its metabolites in the urine of PCOS patients and the control group (Akgül et al., 2019).

#### 3.1.2 Uterine effects

PAEs and their metabolites also playing a crucial role in the formation of endometriosis (Cai et al., 2019). Women with long-term occupational exposure to PAEs show associations with reduced pregnancy rates, increased miscarriage rates, and a significantly elevated risk of implantation failure in women undergoing infertility treatments (Mu et al., 2015).There is a positive correlation between exposure to PAEs or their metabolites and the risk of endometriosis, it can alter the transmission of related signaling pathways to influence the occurrence and development of endometriosis (Kim and Kim, 2020; Sirohi et al., 2021). Huan et al. found that exposure to PAEs, especially DEHP, was associated with the occurrence of endometriosis (Yi et al., 2023). In a meta-analysis, Conforti et al. found that endometriosis was associated with increased urinary levels of MBP/MnBP, MEOHP, and MEHHP, but not for others (Conforti et al., 2021).

### 3.2 Animal experimental research

In animal models, it has been found that exposure to PAEs is associated with a decrease in fertility. More research found that exposure to PAEs resulted in abnormal ovarian function in animal models, but no *in vivo* evidence has shown adverse effects on the endometriosis. Female mice from F1, F2, and F3 generations with uterine exposure to DEHP (0, 0.05, and 5 mg/kg/d) showed reduced oocyte quality and decreased embryonic development ability compared to the control group. Among the F1 female descendants, estrogen levels decreased before estrus, FSH levels increased before and during estrus, and the granulosa cell layer significantly decreased. F3 generation female mice exhibited an overall decrease in body weight, a decrease in pregnancy rate, and an increase in litter size (Meltzer et al., 2015).

PAEs also have adverse effects on the development and maturation of oocytes. DEHP reduces the maturation and fertilization ability of mouse oocytes through impacts on cytoskeletal dynamics of oocytes, oxidative stress, early cell apoptosis, meiotic spindle morphology, mitochondria, ATP content, Juno expression, DNA integrity, and epigenetic modifications (Lu et al., 2019). Another study found that DEHP, by increasing reactive oxygen species levels, disrupting the expression of glutathione peroxidase (GPX), and antioxidant superoxide dismutase 1 (SOD1), induces oxidative stress, leading to the suppression of antral follicle growth (Liu et al., 2021). DEHP can disrupt ovarian function by 17β-HSD signaling pathway, leading to prolonged estrous cycles, increased follicular atresia, inhibition of hormone secretion, decreased expression of steroidogenic enzymes, and promotion of apoptosis in GCs associated with ovarian dysfunction (Li et al., 2020).

DEHP also targets the ovaries through its metabolite mono(2-ethylhexyl) phthalate (MEHP), mediating the impact of DEHP on accelerated follicle development by overactivation the PI3K signaling pathway. Additionally, it inhibits steroidogenesis by reducing the level of steroidogenic enzymes (Hannon et al., 2015). DEHP exposure promotes the generation of reactive oxygen species (ROS) through triggering the CNR1/CRBN/YY1/CYP2E1 signaling axis, inducing DNA damage, cell cycle arrest, and apoptotic cell death in ovarian granulosa cells in mice (Wu et al., 2023).

In summary, PAEs have a structure similar to steroid hormones, which is necessary for their interaction with steroid receptors, and this interaction is associated with endocrine disruption. Female exposure to PAEs can increase the risk of reproductive diseases such as premature ovarian failure, reduced fertility, and adverse pregnancy outcomes. At the hormonal level, PAEs interact with the activity of the HPG axis, which is crucial for normal reproductive development both prenatally and postnatally. Insufficient or excessive hormone levels may lead to reproductive disorders. Additionally, they might act as agonists and antagonists, interfering with nuclear receptors such as AR, ER, and PPAR (Lv et al., 2022). PAEs might also regulate hormone and metabolites balance in the body through the placenta (Fang et al., 2023).

The pervasive use of PAEs and their environmental stability call for an urgent reassessment of their implications on female reproductive health. Future research should prioritize the investigation of the cumulative and synergistic effects of mixed PAE exposures, reflecting the diverse and complex environmental contamination scenarios. Understanding the nuanced mechanisms of PAEs’ reproductive toxicity will enable the development of targeted strategies to mitigate their impact, safeguarding female reproductive health against the insidious effects of environmental pollutants.

## 4 Reproductive toxicity of Triclocarban and Triclosan

Triclocarban (TCC) and Triclosan (TCS) are commonly used antibacterial agents, primarily applied in daily hygiene products such as soap, toothpaste, preservative detergents, as well as various medical disinfectants and toys (Asimakopoulos et al., 2016). TCC and TCS have been identified as globally prominent pollutants and are widely present in rivers, lakes, soil sediments, as well as human blood, urine, amniotic fluid, and placenta (Halden, 2014; Huang et al., 2016). Ingestion, as well as absorption through the skin and mucous membranes, are the primary pathways of exposure to these chemicals.

Currently, the adverse effects of both TCC and TCS on the ecological environment and human reproductive health have garnered public attention. Although TCC and TCS are often discussed and studied together, their chemical structures differ, suggesting they may have distinct biological activities. Both have been confirmed to exert acute and chronic health impacts, including developmental and reproductive toxicity, endocrine-disrupting toxicity, and *in vivo* genotoxicity (Bai et al., 2020).

### 4.1 Epidemiological research

Research confirms that TCC and TCS exhibit estrogenic activity by acting as estrogen receptor agonists through ER, and downregulating the expression of ERα (Huang et al., 2014). In the human body, TCC can be metabolized into sulfate and glucuronic acid conjugates, accelerating absorption and elimination. Currently, there is limited research data on the impact of TCC on human female reproduction. The concentration of TCC in the serum of pregnant women in China ranges from 47.4 to 598 ng/mL, and it has been detected in biological samples such as umbilical cord blood and breast milk. There is evidence suggesting that prenatal exposure to TCC is associated with reduced gestational age. TCC can be transferred to offspring through breastfeeding by mothers exposed to it, reducing offspring survival rate (Kennedy et al., 2015; Enright et al., 2017). The study found that TCC and TCS in maternal and umbilical cord blood are associated with an increased risk of fetal abnormalities (Wei et al., 2017).

TCS is considered a particularly effective competitive inhibitor of mammalian placental estrogen sulfotransferase, which may interfere with the maintenance of pregnancy. Exposure to TCS may have adverse effects in the early stages of human reproduction, and prolonged exposure may lead to decreased fertility (Jurewicz et al., 2020). Research by Du et al. found that TCS exposure disrupts the biosynthesis of steroid hormones and the dynamic balance of hormones, thereby affecting reproductive development (Zhu et al., 2022). TCS has direct or indirect estrogenic effects, possibly by competing with estrogen receptors and disrupting steroidogenic enzymes, disrupting the synthesis of follicle-stimulating hormones, luteinizing hormones, and testosterone, thereby affecting hormone secretion and function (Yoon and Kwack, 2021). The study revealed a significant elevation of TCS levels in the urine of infertile women with PCOS, while in non-PCOS patients, serum TCS levels were positively correlated with luteinizing hormone and the luteinizing hormone/follicle-stimulating hormone ratio (Ye et al., 2018). However, Belén et al. did not observe an association between TCS and infertility; nevertheless, ovarian reserve was affected by TCS (Daza-Rodríguez et al., 2023).

### 4.2 Animal experimental research

TCC has been identified as an endocrine disruptor in many species, affecting embryonic development, body weight, hormones, gene expression, and mouse pup mortality (Rochester et al., 2017). In zebrafish, TCC can stimulate the expression of aromatase, increase estrogen levels, and cause abnormal embryonic development and endocrine disruption (Shi et al., 2019). In a recent study (Costa et al., 2020), pregnant Wistar rats exposed to TCC (0.3, 1.5, and 3.0 mg/kg/day) from gestation day 0 to lactation day 21 showed a decrease in estradiol levels in F1 female offspring. Additionally, in the adult TCC 3.0 mg/kg group, progesterone levels decreased, pre-implantation loss increased, and the reduced synthesis of progesterone may interfere with the implantation process. *In vitro*, exposure of mouse oocytes to TCC showed that TCC disrupts oocyte maturation by affecting the cell cycle process, oxidative stress, early cell apoptosis, cell cytoskeleton dynamics, mitochondrial function, and histone modification (Ding et al., 2020a). Acute exposure to TCC affects early embryonic development, disrupting early embryo gene expression, interfering with ZGA and maternal gene degradation, inducing changes in early embryo epigenetic modifications, leading to oxidative stress and DNA damage in mouse early embryos (Ding et al., 2023). TCC exposure upregulates circSGOL1 in the mother, affecting the activity and localization of hnRNP A1 protein, thereby promoting the overexpression of pro-apoptotic genes, ultimately causing cell apoptosis during early embryo development (Wang et al., 2024). Exposure to TCC *in utero* and during lactation adversely affects follicles and puberty in rat offspring, reducing the number of antral follicles, increasing the atresia index of granulosa cells, inducing cell apoptosis, and disrupting the oxidative/antioxidant balance in the body, affecting the embryo implantation process (Mandour et al., 2021).

*In vitro* studies also found that TCS can impair the proliferation, migration, and dedifferentiation of endometrial stromal cells. Stoker et al. administered different doses of TCS to rats during early and pre-pregnancy, resulting in a significant advancement of vaginal opening time compared to the control group, indicating TCS’s estrogen-like effects (Bitencourt et al., 2019). When mammals are exposed to low concentrations of TCS, it can be absorbed into placental tissue through the placental channel due to the limited chemical barrier function of the placenta (Wang et al., 2015). Furthermore, TCS exposure (10 and 100 mg/kg/day) increased mouse abortion rates and decreased live birth rates. TCS exposure impairs the maturation and early embryo development of porcine oocytes by inducing DNA damage, oxidative stress, and mitochondrial dysfunction (Kim et al., 2020; Park et al., 2020). TCS damages pre-implantation mouse embryo development by triggering the miR-134/Nanog axis, leading to a reduced blastocyst formation rate (Yang et al., 2022). Exposure of zebrafish to TCS revealed that prolonged exposure to TCS can lead to disruption of the endocrine system, resulting in a reduction in the number of normal reproductive cells and a decrease in hatching and survival rates of offspring (Stenzel et al., 2019; Qiao et al., 2022). High doses of TCS induce oxidative damage in zebrafish ovaries and promote reactive oxygen species-dependent cellular apoptosis (Wang et al., 2020; Liu et al., 2022a).

Although there are various studies on the reproductive toxicity of TCC and TCS at different stages, the research focuses vary, and the possible toxic mechanisms are summarized as follows:

Firstly, the structures of TCC and TCS are like non-steroidal estrogen diethylstilbestrol, exhibiting endocrine-disrupting activity. They can interfere with the estrogen signaling system, and enhance the expression of estrogen receptor-responsive genes dependent on estradiol, but do not exhibit estrogenic activity themselves. Secondly, *in vivo*, exposure to TCC and TCS can cause disruptions in substance energy or hormone metabolism, affecting pregnancy outcomes. Thirdly, TCC and TCS may affect placental morphology and maintenance-related gene expression through the placental barrier, thereby influencing fetal development.

Currently, most research on the reproductive toxicity of TCC and TCS in females comes from animal and epidemiological studies. In addition, human exposure pathways and dosage ranges differ from animal studies, making it challenging to establish a direct correlation between the observed negative effects in experimental animals and the decline in human reproductive health. Therefore, it is necessary to conduct comprehensive studies on their reproductive developmental toxicity, deepen the understanding of their toxicological mechanisms, and reduce the harm to female reproductive health.

## 5 Reproductive toxicity of disinfection by-products

Water is the most crucial component of living organisms, and the safety and quality of water are fundamental to human development. Contaminants present in water can harm the reproductive functions of both humans and animals. People use disinfectants in drinking water to kill pathogens, thereby preventing the spread of related diseases. While disinfectants eliminate pathogens, they also undergo various chemical reactions with natural organic matter in water, inevitably producing disinfection by-products (DBPs). Currently, over 700 types of DBPs have been detected (Feng et al., 2019). The diverse categories of DBPs include trihalomethanes (THMs), haloacetonitriles (HANs), haloacetic acids (HAAs), halides (chlorides, chlorates, and bromates), and newly discovered halobenzoquinones (HBQs). Epidemiological studies suggest that populations are widely and continuously exposed to various DBPs, leading to adverse health effects through respiratory, digestive, and skin absorption during prolonged exposure. DBPs exist in the general population, as well as pregnant women, such as blood, urine, and exhaled air (Chen et al., 2019). Some of these DBPs can have detrimental effects on reproduction and exhibit cytotoxicity, genotoxicity, and carcinogenicity (Zuo et al., 2017).

### 5.1 Epidemiological research

DBPs have been demonstrated to be associated with adverse pregnancy outcomes (Säve-Söderbergh et al., 2020; Summerhayes et al., 2021). Iszatt et al. found a significant correlation between exposure to DBPs and a very low birth weight rate (Cao et al., 2016). Rivera et al. observed associations between preterm birth, reduced birth weight, and estimated exposure to DBPs in mid-to-late pregnancy (Iszatt et al., 2014). The study found a negative correlation between urinary DBPs and anti-Müllerian hormone (AMH) and antral follicle count (Deng et al., 2022). In women undergoing assisted reproductive technology treatments, exposure to DBPs in drinking water can lead to changes in reproductive hormone levels, menstrual cycle disruptions, reduced ovarian reserve, inhibition of oocyte maturation, decreased fertilization capacity, and impaired embryo development (Gonsioroski et al., 2020; Liu et al., 2022b; Deng et al., 2023). However, some epidemiological studies have not found a connection between DBP exposure and adverse pregnancy outcomes. A study by Manolis and colleagues, which assessed the exposure levels of DBPs in the water supply area concerning adverse delivery outcomes during pregnancy, found no statistically significant association between exposure levels and preterm birth, very preterm birth, or small for gestational age (SGA) (Kogevinas et al., 2016). Another study by Villanueva and colleagues combined tap water testing data with individual drinking water habits, providing a comprehensive assessment of DBP exposure in pregnant women. The results showed no statistically significant association between DBP exposure and SGA, low birth weight, and preterm birth (Sun et al., 2020).

### 5.2 Animal experimental research

Animal toxicology experiments indicate that DBPs exhibit reproductive toxicity in females, disrupting the production of ovarian steroid hormones and altering reproductive hormone levels. Exposure to DBPs has been shown to significantly inhibit the growth of antral follicles and reduce estradiol levels, resulting in ovarian toxicity. DBPs exposure can lead to developmental and genetic toxicity in zebrafish, significantly reducing tail length and increasing the rate of deformities (Ding et al., 2020b; Chaves et al., 2020). A study using a zebrafish embryo model to assess the developmental toxicity of DBPs found that brominated and iodinated DBPs often have greater toxicity than their chlorinated counterparts. Exposure to halogenated quinones was found to induce the production of ROS in zebrafish embryos, leading to embryonic death, body deformities, DNA oxidative damage, and cell apoptosis (Wang et al., 2018). In addition, *in vivo* experiments in rats, mice, and pigs have also demonstrated the reproductive toxicity of DBPs. Research indicates that exposure to DBPs interferes with the maturation of mouse oocytes by increasing ROS levels, disrupting spindle assembly, inducing DNA damage, and causing meiotic arrest (Jiao et al., 2021).

Exposure to DBPs can affect the function of the HPO axis, significantly inhibiting antral follicle growth and reducing estradiol levels, resulting in ovarian toxicity (Jeong et al., 2016; Gonzalez et al., 2021). Prenatal and lactational exposure to DBPs affects mouse vaginal opening, anogenital distance, ovarian weight, percentage of atretic follicles, and hormone levels in the F1 generation (Gonsioroski et al., 2022). Exposure to environmentally relevant concentrations of bromodichloromethane can induce changes in the transcriptome and epigenome, adversely affecting blastocyst formation rates and alterations in the estradiol pathway (Pagé-Larivière et al., 2016). Although some studies suggest an association between DBPs and adverse reproductive outcomes, Narotsky et al. did not observe any impact on fertility, pregnancy maintenance, prenatal survival, postnatal survival, or birth weight in parent, F1, and F2 generations of rats exposed to a regulated mixture of DBPs (Narotsky et al., 2015).

The inconsistencies between epidemiological research and animal experiments can be attributed to several factors: 1. Differences in Exposure Assessment: Discrepancies may arise due to variations in the methods used to assess exposure. Multiple sampling occasions may be required to enhance the accuracy of exposure assessment. 2. Variability in Experimental Animals: The species, strains, and individual differences among experimental animals can impact the reproducibility and uniformity of experimental results.

As DBPs are widely present pollutants in drinking water, the long-term risks associated with their exposure to reproductive health remain a subject of significant concern. While both toxicological and epidemiological studies suggest that higher concentrations of DBPs may pose a risk to fetal growth and development, the specific mechanisms are still under investigation. Therefore, conducting additional toxicological and epidemiological research is crucial. This research can provide a better understanding of the potential harm to reproductive health caused by long-term exposure to DBPs, offering a theoretical basis for the prevention of reproductive diseases.

## 6 Summary and prospect

EDCs are widely distributed and can directly or indirectly affect the female reproductive system, impairing development and fertility. EDCs can cause damage to the reproductive organs such as the pituitary gland, ovaries, and uterus during follicle development, embryonic formation, puberty, and reproductive periods, leading to reproductive dysfunction and the occurrence of related diseases. This article reviews the research progress on the relationship between five major EDCs and female reproduction, assessing their impact on female reproductive potential, including irregular estrous cycles, hormonal disruptions, reduced follicle numbers, decreased ovarian function, reduced pregnancy rates, and lower offspring survival rates. EDCs exhibit biological effects similar to endogenous hormones in the body and can generate toxic effects through the activation of receptors such as estrogen receptors, aryl hydrocarbon receptors, and peroxisome proliferator-activated receptors. Delving deeper into the molecular mechanisms of reproductive toxicity related to specific EDCs exposure presents a critical area for future research. Furthermore, identifying particular cellular signaling pathways or biomarkers to ascertain the effects of EDCs on the female reproductive system is indeed paramount for advancing our understanding and developing targeted interventions.

Further research is needed to explore the direct effects of EDCs on the hypothalamus, pituitary gland, ovaries, and uterus, as these organs regulate female fertility and are associated with the onset of reproductive aging. However, not all studies have reported a significant association between exposure to chemical substances and adverse reproductive outcomes in humans. This may be related to differences in study populations, sample sizes, methods of measuring exposure levels, and the high variability in measured reproductive outcomes between studies.

Additionally, most studies have only reported the reproductive toxicity effects of individual substances, without considering the potential interactions between different substances. Over the past decade, the impacts of exposure to various chemicals on human health have garnered increasing attention. In the realm of statistics, methodologies such as Weighted Quantile Sum Regression (Carrico et al., 2015), Bayesian Kernel Machine Regression (Bobb et al., 2015), and Quantile-Based g-Computation (Keil et al., 2020) have been continuously refined, facilitating the evaluation of combined effects from multiple exposures. To date, only a few of studies have focused on the effects of PAEs mixtures on ovarian function, including aspects such as ovarian cycle, hormone levels, and implications for fertility rates (Laws et al., 2023; Safar et al., 2023). The combined effects of exposure to multiple chemicals on female fertility warrants further investigation.

Few studies have evaluated risk factors that may affect the concentration of such substances in the female reproductive system and other reproductive parameters. The impact of EDCs on women’s reproductive health needs to consider the influence of other substances and lifestyle factors, as the toxicity of different substances depends on exposure levels, duration, exposure routes, types of toxins, workplace conditions, medical history, and other physiological factors. Further clarification of the exact mechanisms of reproductive toxicity caused by EDCs will provide a theoretical basis for preventing and treating reproductive diseases.
